# Intra-articular Migration of a Broken Needle in an Intravenous Drug User: A Case Report and Surgical Management

**DOI:** 10.7759/cureus.92943

**Published:** 2025-09-22

**Authors:** Rahul Kaushik, Taruna Singh, Devinder Kumar, Labhanshi Aggarwal, Mohd Altaf Mir

**Affiliations:** 1 Burns and Plastic Surgery, All India Institute of Medical Sciences, Bathinda, Bathinda, IND

**Keywords:** broken needle, foreign body migration, heroin injection, intra-articular retrieval, intravenous drug user, knee joint

## Abstract

Intravenous drug users (IVDUs) frequently present with complications; however, intra-articular migration of a broken needle is exceedingly rare.

This is the case of a 29-year-old male heroin user who experienced needle breakage during self-injection into the popliteal fossa. The initial removal attempts failed, and he presented with knee discomfort 10 days later. Radiographs revealed a metallic foreign body in the popliteal fossa, near the femur. Posterior surgical exploration failed to locate the needle. Intraoperative imaging showed anterior migration. A medial parapatellar arthrotomy was performed, and the needle was retrieved between the anterior cruciate ligament (ACL) and the lateral meniscus. This case highlights the importance of intraoperative imaging and surgical flexibility. Early diagnosis and retrieval are crucial for avoiding joint damage and complications.

## Introduction

Intravenous drug use (IVDU) is a growing public health concern associated with numerous local and systemic complications ranging from soft tissue infections and thrombophlebitis to endocarditis and pulmonary embolism. A less commonly recognized but potentially serious complication is the breakage and retention of the injection needles during self-administration. While most retained needles remain in the subcutaneous or muscular tissues, some may migrate, posing diagnostic and therapeutic challenges [1,2].

Needle breakage is often underreported because of social stigma, lack of awareness, or asymptomatic presentation [2]. However, retained metallic foreign bodies may cause pain, infection, or embolization, and breakage is often due to collapse after sudden depression of consciousness due to the narcotic. Cases of intravascular migration leading to pulmonary embolism and cardiac complications have been reported [3,4]. Rarely, broken needles may traverse anatomical planes or joints and enter intra-articular spaces. The literature on intra-articular migration of foreign bodies, especially broken needles, in IVDUs is sparse. To date, such migration has been reported more frequently in the context of orthopedic implants, such as tension band wires or interference screws, which may migrate into the knee joint or surrounding tissues [5-7].

The diagnostic localization of small metallic fragments is often difficult. While plain radiographs are typically used initially, three-dimensional computed tomography (3D-CT) or intraoperative fluoroscopy may be required for precise localization, especially when migration has occurred [8]. In such scenarios, flexible surgical planning and intraoperative decision-making are critical for safe retrieval and joint preservation.

We present a rare case of intra-articular migration of a broken needle following attempted intravenous heroin injection in the popliteal fossa, emphasizing diagnostic and surgical considerations in managing such patients.

## Case presentation

A 29-year-old male with a known history of intravenous heroin use presented to our outpatient department (OPD) with localized discomfort in the left knee. Ten days prior to presentation, the patient attempted to self-inject heroin intravenously in the popliteal fossa area using a hypodermic needle that broke during insertion. An unsuccessful attempt at foreign body removal was made at an outside facility. The patient reported progressive discomfort around the posterior aspect of the right knee but denied systemic symptoms such as fever or chills.

On physical examination, the left knee showed moderate effusion and localized tenderness over the posterior aspect. A previous linear incision of approximately 4 cm was observed in the popliteal fossa of the previous attempt at removal. The knee range of motion was mildly restricted due to pain, particularly in deep flexion, but the distal neurovascular status remained intact.

**Figure 1:**
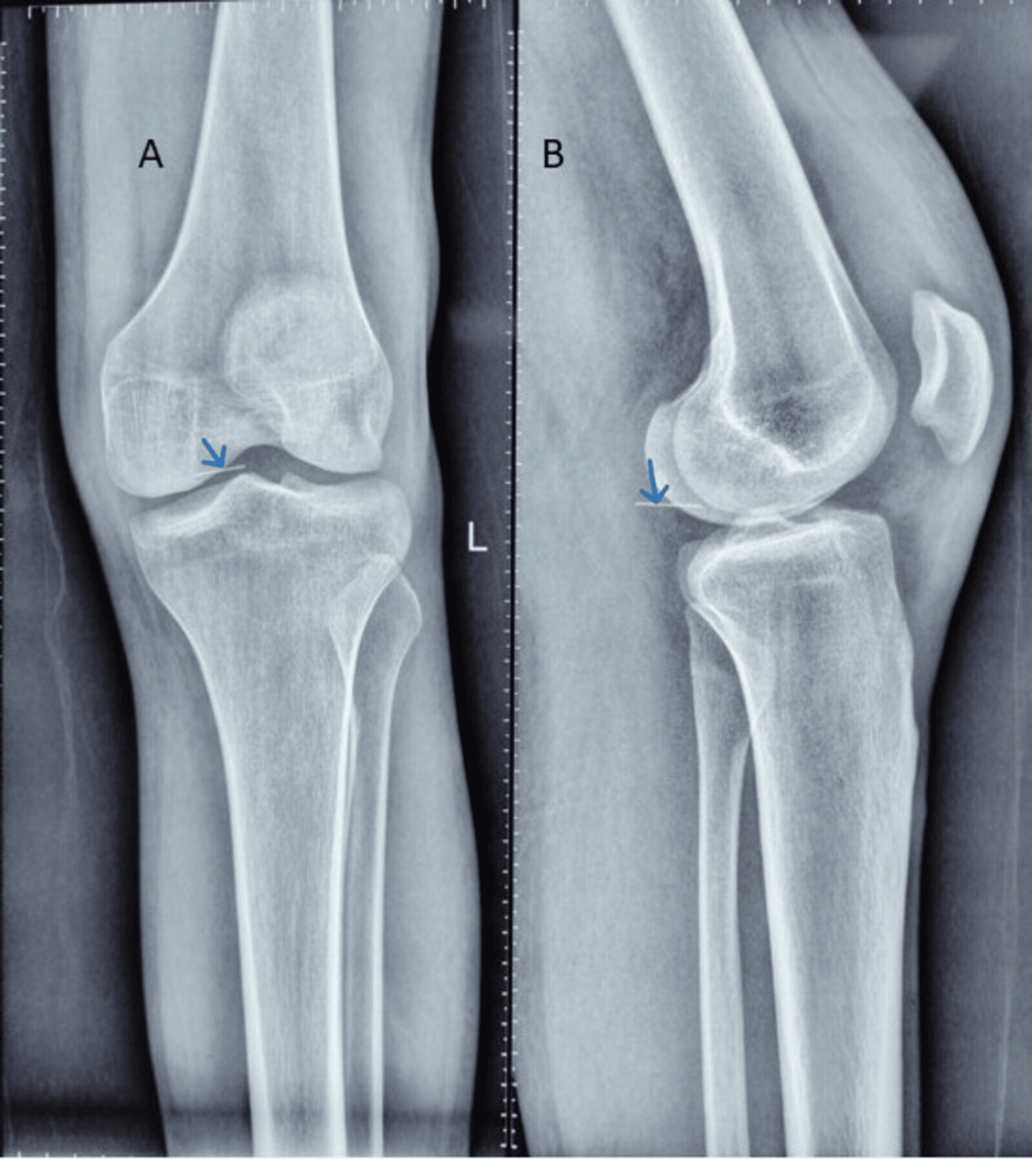
Preoperative radiograph Preoperative radiograph (Panel A: anteroposterior view and Panel B: lateral view) obtained on the day of surgery shows a broken needle (arrow) in the popliteal fossa close to the medial femoral condyle.

Radiographs of the knee revealed a linear metallic foreign body in the popliteal region, appearing close to the posterior aspect of the distal femur (Figure 1).

Routine blood tests were within normal limits, and there were no clinical or laboratory indicators of systemic infection.

**Figure 2:**
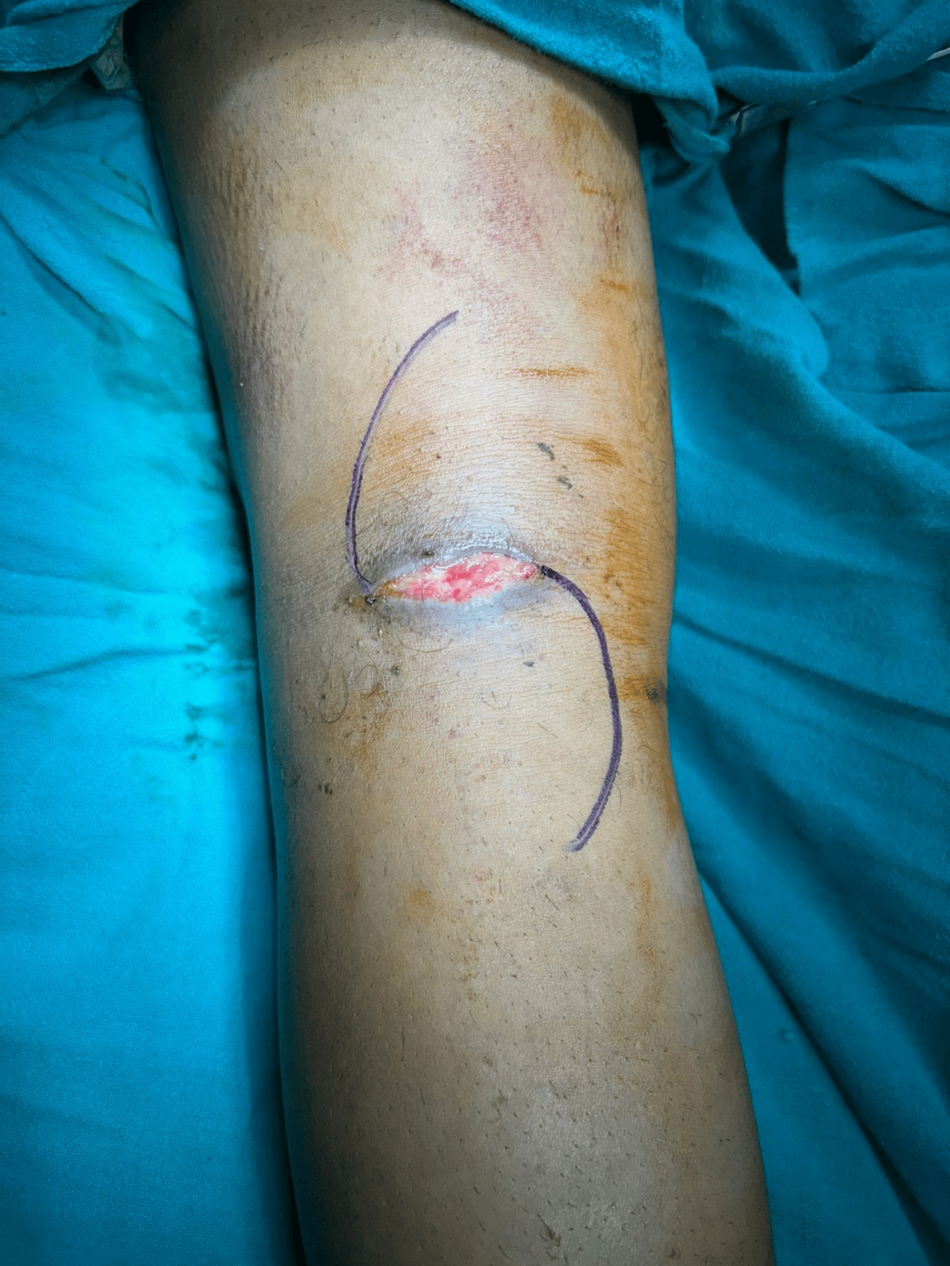
Intraoperative image of incision Intraoperative image showing the previous incision site, which was strategically incorporated into the new surgical approach to minimize additional soft tissue trauma.

Given the radiographic findings and history, the patient underwent surgical exploration under general anaesthesia. A posterior approach to the knee was used to access the presumed location of the foreign body (Figure 2).

**Figure 3:**
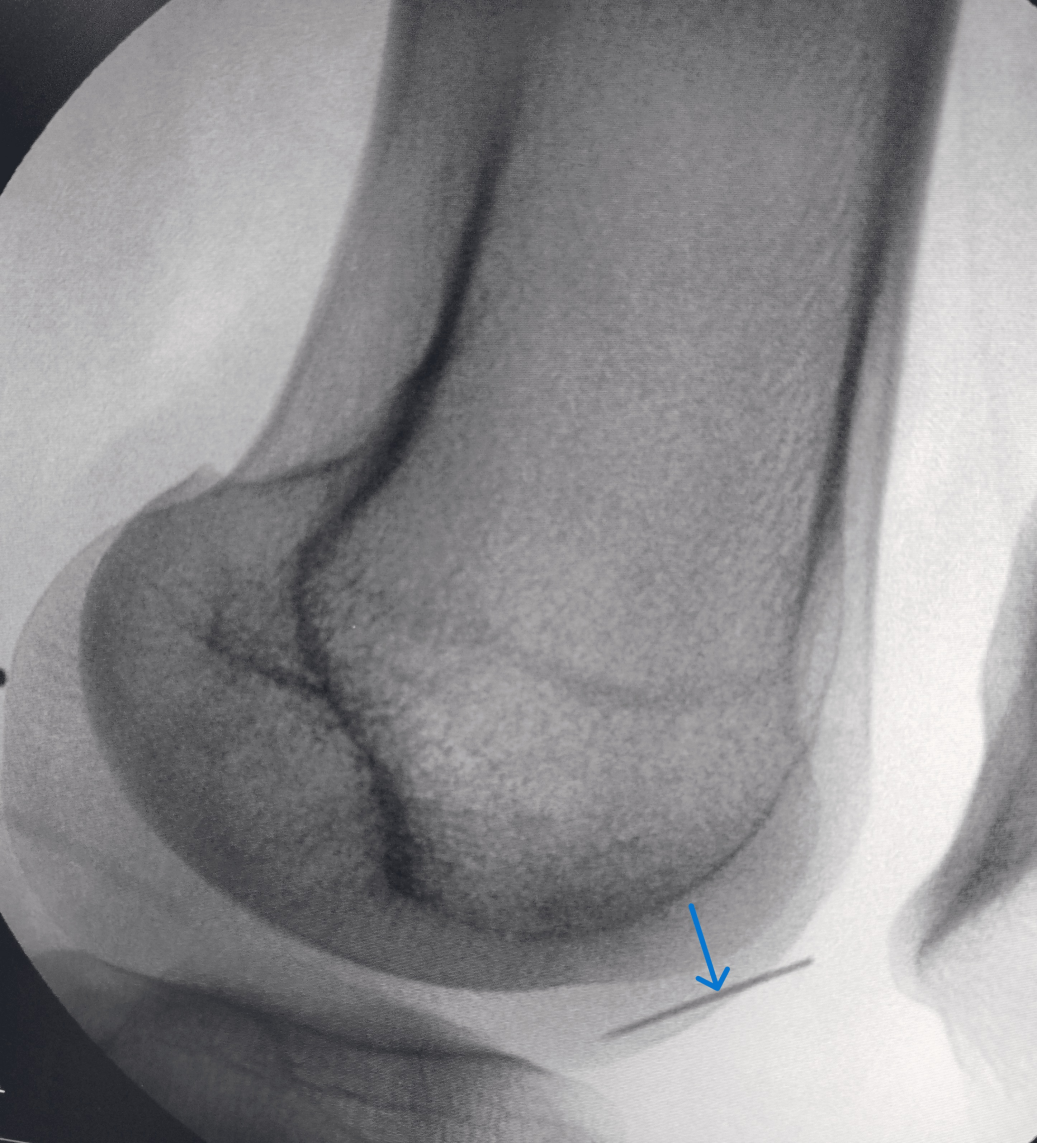
Intraoperative fluoroscopic image of the left knee (lateral view) Intraoperative fluoroscopic image demonstrating anterior migration of the broken needle (indicated by an arrow).

Despite a thorough exploration of the popliteal fossa, the needle could not be located. Intraoperative fluoroscopy revealed that the needle had migrated anteriorly within the joint cavity (Figure 3).

**Figure 4:**
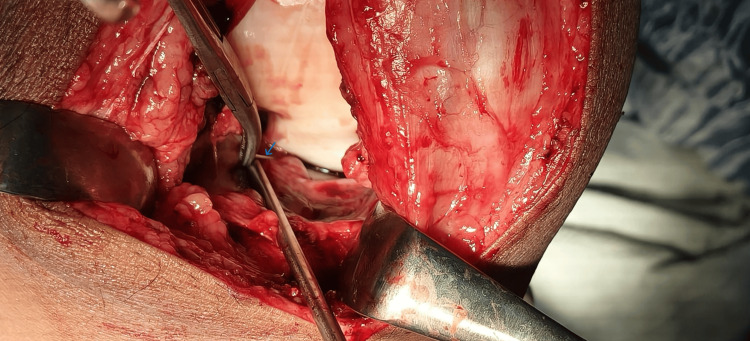
Intra-operative picture of needle localization The picture depicts a needle lying (as shown by the arrow) in a narrow space between the anterior cruciate ligament and the lateral meniscus.

Accordingly, the surgical plan has been modified. The patient’s position was changed from prone to supine, and a medial parapatellar arthrotomy was performed (Figure 4).

**Figure 5:**
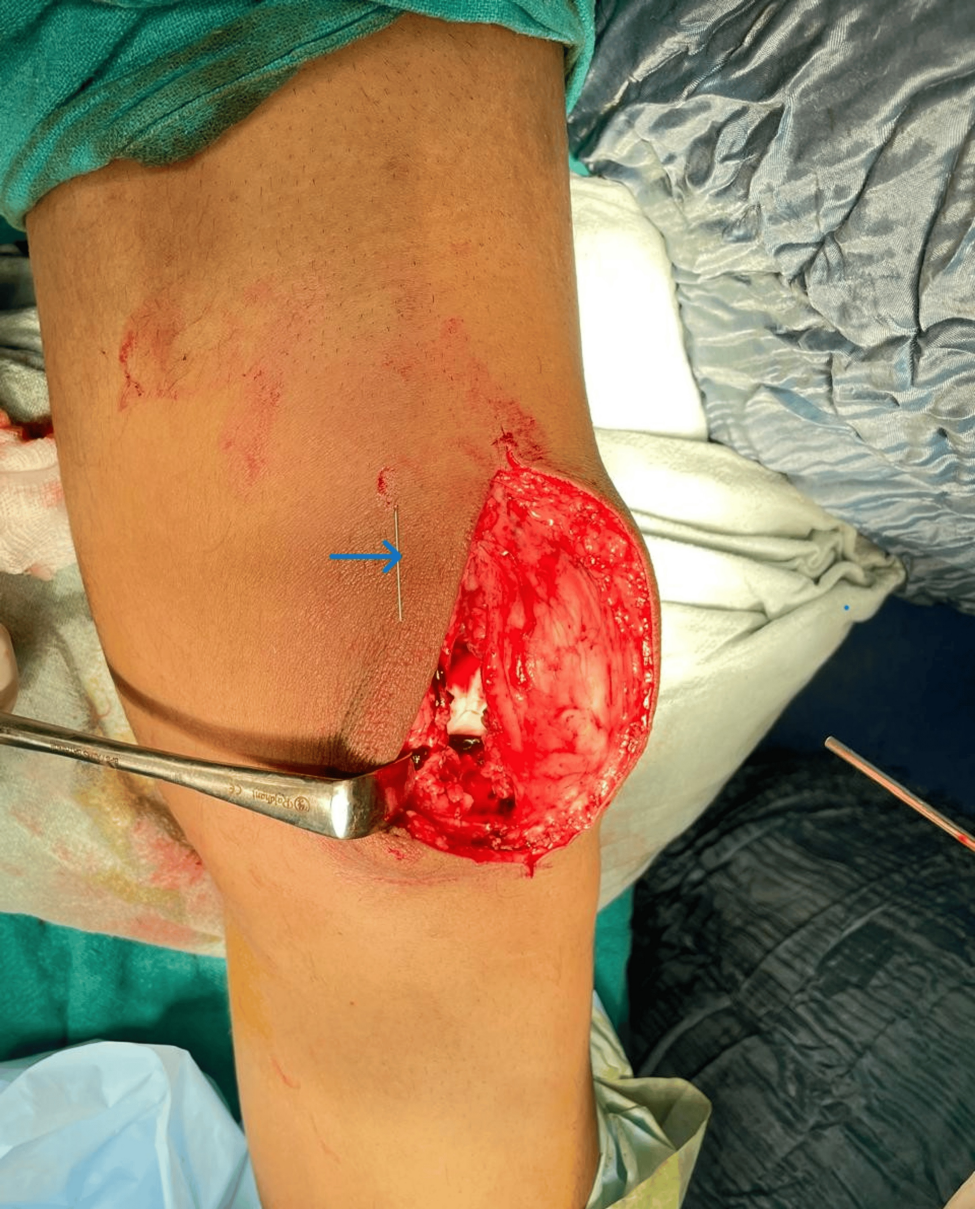
Intra-operative image showing the retrieved needle Photograph showing the retrieved 26G needle, 4 cm in length (shown by an arrow), from the anterior approach after migration from posterior to anterior of the knee.

The broken needle was identified as lodged between the anterior cruciate ligament and lateral meniscus and was successfully removed without further damage to the intra-articular structures (Figure 5).

The patient recovered uneventfully postoperatively. He was started on a short course of antibiotics and discharged on the third postoperative day. Prior to discharge, counseling for substance abuse and harm reduction strategies was initiated, and the patient was referred to a de-addiction specialist.

## Discussion

This case highlights several important considerations in the management of retained and migrating foreign bodies, particularly in the context of intravenous drug abuse. Although needle breakage is often seen among chronic users owing to the repeated use of fragile or damaged syringes, intra-articular migration is exceedingly rare [1,2].

In our patient, the popliteal fossa was the initial site of needle breakage. This anatomical region is rich in neurovascular structures and is adjacent to the posterior knee joint capsule. Although the initial radiograph showed that the needle was close to the posterior femur, its subsequent anterior migration likely occurred because of joint movement or synovial fluid currents. Similar cases of implant migration into the knee joint have been reported, including the migration of broken tension band wires and ACL graft components [5,6]. These cases, similar to ours, necessitated exposure through arthroscopy; however, we preferred arthrotomy for foreign body retrieval.

Failure of posterior exploration and the eventual need for medial parapatellar arthrotomy highlight the necessity for intraoperative imaging. Intraoperative fluoroscopy has proven to be an invaluable tool for localizing radiopaque foreign bodies during surgery, especially when migration has occurred [9]. Preoperative 3D-CT imaging has also been utilized effectively in other cases to aid the precise localization of metallic fragments, reducing operative time and unnecessary dissection [8]. However, we preferred intraoperative fluoroscopy for the localization of foreign bodies. The fluoroscopy for the localization of foreign bodies can be attempted just before planning the incision, which will minimize the duration of the operative procedure.

Our case also draws parallels with previously reported cases of pulmonary or vascular embolization of broken needles. Arafat et al. documented a case of needle embolism into the pulmonary artery in a drug user, emphasizing that once within the vascular system, such foreign bodies can travel far from their entry point and cause significant morbidity [3]. Although our case was not vascular, it involved unpredictable migration, underscoring the importance of thorough assessment and imaging.

Furthermore, unrecognized or retained foreign bodies pose risks of chronic synovitis, cartilage damage, infection, and joint dysfunction if left untreated [7]. Therefore, timely diagnosis and retrieval are essential to preserve joint integrity and function.

This case highlights the psychosocial aspects of care in IVDUs. Singh et al. emphasized the underreporting and concealment of broken needle incidents due to the fear of legal repercussions or social stigma [2]. A high index of suspicion is required in such cases, and healthcare providers must approach these patients with non-judgmental empathy to ensure timely and effective treatment.

## Conclusions

This case highlights the rare occurrence of the intra-articular migration of a broken hypodermic needle in an intravenous drug user, initially localized in the popliteal fossa but later found lodged between the ACL and lateral meniscus. It emphasizes the importance of considering foreign body migration when retrieval attempts fail, and the critical role of intraoperative imaging in accurate localization. Flexibility in surgical planning, along with timely intervention, is essential to ensure safe removal, prevent further joint damage, and preserve knee function in such challenging scenarios.
